# Histamine 1 receptor-G_βγ_-cAMP/PKA-CFTR pathway mediates the histamine-induced resetting of the suprachiasmatic circadian clock

**DOI:** 10.1186/s13041-016-0227-1

**Published:** 2016-05-06

**Authors:** Yoon Sik Kim, Young-Beom Kim, Woong Bin Kim, Seung Won Lee, Seog Bae Oh, Hee-Chul Han, C. Justin Lee, Christopher S. Colwell, Yang In Kim

**Affiliations:** Department of Physiology and Neuroscience Research Institute, Korea University College of Medicine, 126-1 Anam-dong 5-ga, Seoul, 136-705 Republic of Korea; Pain Cognitive Function Research Center, Dental Research Institute and Department of Neurobiology and Physiology, Seoul National University, Seoul, 110-749 Republic of Korea; Center for Neuroscience and Functional Connectomics, Korea Institute of Science and Technology, Seoul, 136-791 Republic of Korea; Department of Psychiatry & Biobehavioral Sciences, University of California-Los Angeles, 760 Westwood Plaza, Los Angeles, CA 90024 USA

**Keywords:** Calcium, CFTR, Chloride, Circadian rhythm, Histamine, NKCC1, Suprachiasmatic nucleus

## Abstract

**Background:**

Recent evidence indicates that histamine, acting on histamine 1 receptor (H1R), resets the circadian clock in the mouse suprachiasmatic nucleus (SCN) by increasing intracellular Ca^2+^ concentration ([Ca^2+^]_i_) through the activation of Ca_V_1.3 L-type Ca^2+^ channels and Ca^2+^-induced Ca^2+^ release from ryanodine receptor-mediated internal stores.

**Results:**

In the current study, we explored the underlying mechanisms with various techniques including Ca^2+^- and Cl^−^-imaging and extracellular single-unit recording. Our hypothesis was that histamine causes Cl^−^ efflux through cystic fibrosis transmembrane conductance regulator (CFTR) to elicit membrane depolarization needed for the activation of Ca_V_1.3 Ca^2+^ channels in SCN neurons. We found that histamine elicited Cl^−^ efflux and increased [Ca^2+^]_i_ in dissociated mouse SCN cells. Both of these events were suppressed by bumetanide [Na^+^-K^+^-2Cl^−^ cotransporter isotype 1 (NKCC1) blocker], CFTR_inh_-172 (CFTR inhibitor), gallein (G_βγ_ protein inhibitor) and H89 [protein kinase A (PKA) inhibitor]. By itself, H1R activation with 2-pyridylethylamine increased the level of cAMP in the SCN and this regulation was prevented by gallein. Finally, histamine-evoked phase shifts of the circadian neural activity rhythm in the mouse SCN slice were blocked by bumetanide, CFTR_inh_-172, gallein or H89 and were not observed in NKCC1 or CFTR KO mice.

**Conclusions:**

Taken together, these results indicate that histamine recruits the H1R-G_βγ_-cAMP/PKA pathway in the SCN neurons to activate Ca_V_1.3 channels through CFTR-mediated Cl^−^ efflux and ultimately to phase-shift the circadian clock. This pathway and NKCC1 may well be potential targets for agents designed to treat problems resulting from the disturbance of the circadian system.

## Background

Histamine, a neurotransmitter/neuromodulator produced by tuberomammillary nucleus (TMN) neurons in the hypothalamus [1], has a crucial role in regulating brain arousal [2]. A large body of evidence indicates that histamine is also a potent regulator of the mammalian circadian time-keeping system. Histaminergic fibers from the TMN densely innervate the master circadian clock suprachiasmatic nucleus (SCN) in the rat [3–5] and exogenously applied histamine exerts a phase-shifting effect on the circadian neural activity rhythms recorded in the hamster and mouse SCN slices maintained *in vitro* [6, 7] and on the circadian behavior rhythms manifested in rats [8]. Moreover, histamine synthesis inhibition disrupts circadian activity rhythms in the rat [8] and reduces the phase shifts of circadian activity rhythms induced by light in the hamster [9].

It is well established that 4 different types of histamine receptors (i.e., H1R ~ H4R) mediate histaminergic actions and that the H1R, which is linked to the G_q/11_-phospholapase C (PLC) pathway, mediates a majority of excitatory actions of histamine in the central nervous system [2, 10]. The G_q/11_-PLC pathway leads to diacyl-glycerol (DAG) and inositol 1,4,5-trisphosphate (IP_3_) production [2, 10]. While DAG can cause Ca^2+^ influx through the transient receptor potential cation channel (TRPC), IP_3_ induces Ca^2+^ release from the internal stores via IP_3_ receptor [10, 11].

Recently, we demonstrated that histamine, acting on H1R, increases intracellular Ca^2+^ concentration ([Ca^2+^]_i_) in mouse SCN neurons by a novel mechanism driven by Ca_V_1.3 L-type Ca^2+^ channels as well as Ca^2+^-induced Ca^2+^ release from ryanodine receptor (RyR)-mediated internal stores and this is the molecular mechanism underlying the histamine-induced phase delay of circadian neural activity rhythm in the SCN [12]. In the present study, we sought to delineate the novel signaling mechanisms leading to L-type Ca^2+^ channel opening after H1R activation. Prior works have shown that histamine regulate Cl^−^ conductances in neurons [13, 14] and that, in some SCN neurons, the electrochemical gradient for Cl^−^ is set toward the extracellular side due to the Cl^−^-importing activity of Na^+^-K^+^-2Cl^−^ cotransporter isotype 1 (NKCC1) [15, 16]. Therefore, we hypothesized that histamine causes Cl^−^ efflux to elicit membrane depolarization needed for L-type Ca^2+^ channel activation. We considered the possibility that the cystic fibrosis transmembrane conductance regulator (CFTR) may mediate the histamine-induced Cl^−^ efflux because Allen Brain Atlas ([17], http://www.brain-map.org) indicates the presence of CFTR mRNA in the mouse SCN. Prior works also indicate that H1R is positively coupled through the G_βγ_ protein to the cAMP signaling pathway [18, 19], which can lead to CFTR activation by stimulating protein kinase A (PKA) [20–24]. Here we present results indicating that the G_βγ_-cAMP/PKA-CFTR pathway links H1R to L-type Ca^2+^ channels and this pathway is essential for the phase delay of the circadian clock induced by histamine.

## Methods

### Study approval

The procedures of experiments employed in the current study were approved by the Animal Research Policies Committees of Korea University College of Medicine and Korea Institute of Science and Technology. Also, they conformed to the guidelines of National Institutes of Health of the United States of America.

### Animals and housing

Male C57BL/6 mice (B6 mice, 3–6 week-old) bred in Korea Institute of Science and Technology were used for Ca^2+^ imaging, Cl^−^ imaging, enzyme immunoassays (EIA) and extracellular single-unit recording experiments. In addition, male knock-out (KO) mice (3–6 week-old) for NKCC1 (Slc12a2^−/−^) or CFTR (B6.129P2-Cftr^tm1Unc^/J), obtained from Professor Min Goo Lee at Yonsei University (Seoul, Korea), were used in some of these experiments. Before being used, the mice were housed in group cages (4–6/cage) in a vivarium (22–24 °C) with a 12 h/12 h light/dark cycle for ≥ 1 week. The times of lights-on and -off in the vivarium were designated as zeitgeber time (ZT) 0:00 h and ZT 12:00 h, respectively.

### Preparation of brain slices

Brain slices were prepared between ZT 10:00 h and ZT12:00 h. Under urethane anesthesia (1.25 g/kg, i.p.), the brain was quickly resected and put in aerated (95 % O_2_/5 % CO_2_) ice-cold artificial cerebrospinal fluid (ACSF), which was composed of (in mM) 124 NaCl, 1.3 MgSO_4_, 3 KCl, 1.25 NaH_2_PO_4_, 26 NaHCO_3_, 2.4 CaCl_2_ and 10 glucose. After being chilled in the ACSF for 1–2 min, the brain was cut into slices (300–400 μm thickness) in ice-cold ACSF using a vibroslicer (World Precision Instruments or Leica VT100S). The slices containing the SCN were selected and kept in aerated (95 % O_2_/5 % CO_2_) ACSF at room temperature (R/T, 30–60 min) before being used for SCN cell dissociation or being transferred to an electrophysiological recording chamber, which was continuously perfused with aerated (95 % O_2_/5 % CO_2_) ACSF (35 °C).

### SCN cell dissociation

The hypothalamic slices incubated in aerated ACSF as above were used for SCN cell dissociation. The procedures for the cell dissociation were identical to those described previously [12].

### Ca^2+^ imaging

The Ca^2+^ imaging methods employed in this study were identical to those described previously [12]. [Ca^2+^]_i_ was expressed as the ratio of intensities of the fura-2 emission fluorescence of 510 nm, excited at 340 and 380 nm. All the Ca^2+^-imaging experiments (as well as Cl^−^-imaging study, below) were carried out between ZT 13:00 h and ZT 18:00 h.

### Cl^−^ imaging

To measure changes of intracellular Cl^−^ concentrations ([Cl^−^]_i_), the Cl^−^ sensor N-(ethoxy carbonyl methyl)-6-methoxyquinolinium bromide (MQAE, Invitrogen, CA) was used. This sensor shows the highest fluorescence in the absence of Cl^−^, and therefore, a decrease of the monitored fluorescence represents an increase in [Cl^−^]_i_, and vice versa. Dissociated SCN cells plated on poly-D-lysine-coated glass coverslips were incubated for 2 h in HEPES-buffered saline that contained 2-mM MQAE and then rinsed with HEPES-buffered saline before being moved to an imaging chamber mounted on an inverted microscope (IX70, Olympus). MQAE was excited at 350 nm and emitted at 460 nm. Imaging data were acquired with the use of an intensified CCD camera (CasCade, Roper Scientific) and Metafluor software (Molecular Device). Changes in [Cl^−^]_i_ were calculated as ΔF with

$$ \Delta \mathrm{F}=\frac{\left(\mathrm{F}\hbox{-} \mathrm{F}0\right)}{\mathrm{F}0} $$

where F is the fluorescence at each time point and F0 is the average of the fluorescence measured at the first ten time points.

### Microdissection of the SCN and cAMP measurement

Brain slices (350-μm thickness) were prepared from B6 mice (4 ~ 5 week-old) as described above. After being treated with drugs (see Results) for 30 min starting at ZT 14:00 h, the slices were mounted on a silicon rubber stage chilled with dry-ice powder. The SCN on both the left and right sides were punched out from the frozen section with the use of a blunt needle (21 G) under a dissecting microscope. The SCN punches from each hypothalamic slice were pooled in 0.1-N HCl solution (300 μl) and then sonicated with Bioruptor (COSMO BIO, Japan) at 4 °C. The concentration of cAMP was determined with the use of Cyclic AMP EIA kit (Cayman Chemical, MI) according to the manufacturer’s protocol. The protein content was determined with Pierce™ BCA Protein Assay Kit (Thermo scientific, MA). Following centrifugation at 1,000 *g* for 10 min, the supernatant was diluted 1:2 with EIA buffer (Cayman Chemical). All SCN samples were acetylated prior to the determination of cAMP level by EIA. The assays were performed in duplicate, with measurement at λ = 405 nm on a micro plate reader (SPECTRA max plus 384, Molecular Devices, CA).

### Experimental treatment and single-unit recording

On the first day, the slice was treated with histamine for 30 min from ZT 14:00 h. In certain experiments, histamine was applied to the slice along with bumetanide (NKCC blocker), gallein (G_βγ_ blocker), CFTR_inh_-172 (CFTR blocker), H89 (PKA blocker) or KT5720 (PKA blocker). The application of these drugs commenced at ZT 13:50 h and lasted for 50 min. Control slices were challenged neither with histamine nor with any of the blockers above.

On the second day, single-unit recordings were obtained extracellularly from the SCN at ZT 1:00–15:00 h. To detect the time at which the peak of circadian firing activity rhythm of SCN neurons occurs (i.e., a reliable marker of the phase of circadian clock [25]), we plotted against ZT the average firing rates of randomly sampled single units for sequential 2-h periods with 1-h lags [25]. To quantify the phase shift resulting from a drug treatment, we measured the difference of the time-of-peak found in the experimental slice from the average time-of-peak of control slices [12, 25].

### Drugs

Drugs and chemicals utilized in the present study were from Sigma-Aldrich (St. Louis, MO) or Tocris Bioscience (Bristol, UK). The solutions of histamine (100 μM; [26–28]), 2-pyridylethylamine (100 μM; [29–33]) and amthamine (20 μM; [34–36]) were prepared in ACSF or HEPES-buffered saline. The solutions of bumetanide (10 μM; [16, 37, 38]), CFTR_inh_-172 (50 μM; [39–41]), gallein (100 μM; [42–44]), H89 (10 μM; [45–47]), KT5720 (1 μM; [48–50]), bicuculline (30 μM; [51–53]) and strychnine (1 μM; [54–56]) were prepared by diluting the stock solutions with ACSF or HEPES-buffered saline; the solvent of stock solutions was dimethyl sulphoxide and its final concentration was 0.01–0.003 %. The drug solutions were applied to SCN slices or dissociated SCN cells by peristaltic pump or gravity-fed bath-perfusion system [15].

### Statistics

Numerical data are expressed as the mean ± SEM. Student *t*-test and Mann–Whitney Rank Sum test were employed to compare two independent data sets with and without normal distribution, respectively. Paired samples having normal distribution were compared with paired *t*-test while paired samples without normal distribution with Wilcoxon Signed Rank test. One-way analysis of variance (ANOVA) and pairwise comparison with Student-Newman Keuls tests were performed to compare ≥3 independent data sets having normal distributions, while Kruskal-Wallis one-way ANOVA on ranks and pairwise comparison with Student-Newman Keuls tests were performed to compare ≥ 3 data sets lacking normal distributions. Chi-square test was used to determine whether there is a significant difference between the expected frequencies and the observed frequencies in one or more categories. *P* < 0.05 (two-sided) was considered significant.

## Results

### Histamine induces the efflux of NKCC1-accumulated Cl^−^ and, through this mechanism, activates L-type Ca^2+^ channels in SCN neurons

**Fig. 1:**
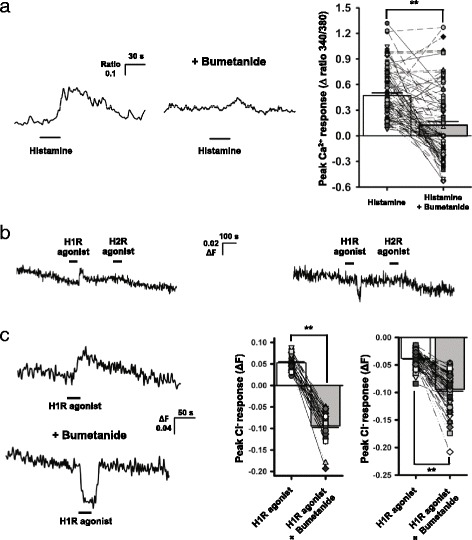
Histamine induces the efflux of NKCC1-accumulated Cl^−^, leading to intracellular Ca^2+^ rise in SCN neurons. (**a**, *left panel*) Traces from a Ca^2+^-imaging experiment which show the effect of bumetanide on histamine-elicited increase in [Ca^2+^]_i_ in an SCN neuron. (**a**, *right panel*) Graphs summarizing the effects of bumetanide on the histamine-elicited Ca^2+^ responses in 105 SCN neurons from 3 mice. The bar charts indicate the mean (± SEM) peak Ca^2+^ responses. **b** Traces from Cl^−^-imaging experiments show the effects of H1R agonist on [Cl^−^]_i_ in SCN cells. Up- and downward deflections of the trace denote decrease and increase in [Cl^−^]_i_, respectively. **c** Traces and summary graphs showing the effects of bumetanide on H1R agonist-induced decrease (*n* = 81) and increase (*n* = 80) in [Cl^−^]_i_ in SCN cells. The bar charts indicate the mean (± SEM) peak Cl^−^ responses. The symbols connected by lines in (**a**) and (**c**) denote data from the same cells. **: *p* < 0.001, paired *t*-test

To test our hypothesis, we first examined how the NKCC inhibitor bumetanide affects the histamine (100 μM, 30 s)-elicited rise in [Ca^2+^] in dissociated SCN neurons and whether NKCC1 KO mouse neurons would show an altered histamine response. Bumetanide (10 μM) attenuated the histamine-elicited Ca^2+^ rise in 41 of 105 cells (0.54 ± 0.06 to 0.20 ± 0.05) or converted the rise into fall in 40 of 105 cells (0.47 ± 0.04 to −0.31 ± 0.12) (Fig. 1a). In the remaining cells, it did not affect (7 of 105 cells; 0.77 ± 0.09 *vs*. 0.78 ± 0.09) or enhanced the histamine-elicited Ca^2+^ increase (17 of 105 cells; 0.32 ± 0.06 to 0.61 ± 0.08) (Fig. 1a). These effects of bumetanide were statistically significant [t(104) = 8.302, *p* < 0.001, paired *t*-test]. Likewise, histamine-evoked Ca^2+^ response was reduced in SCN neurons prepared from NKCC1 KO mice. In the KO mice, histamine application caused Ca^2+^ rise in 31 % of the neurons (28 of 90 cells from 2 mice) with an average increase of 0.30 ± 0.04 (Δ ratio 340/380). In wild-type mice, histamine application resulted in an increase in Ca^2+^ level in 60 % of the neurons (116 of 192 cells from 3 mice) with an average increase of 0.50 ± 0.03 (Δ ratio 340/380). Thus, in NKCC1 KO mice, the proportion of cells showing histamine-induced Ca^2+^ response was significantly reduced [Chi-square(2) = 44.282, *p* < 0.001, Chi-square test] as well as the average magnitude of the response [U = 2120, *p* < 0.001, Mann–Whitney Rank Sum test]. Taken together, these results support the hypothesis that histamine induces the efflux of NKCC1-accumulated Cl^−^ in at least a subset of SCN neurons and through that mechanism increases [Ca^2+^]_i_.

In order to obtain more direct evidence that histamine causes Cl^−^ efflux in SCN neurons, we next performed Cl^−^-imaging experiments using the fluorescent Cl^−^ sensor MQAE. Bath application of the H1R agonist 2-pyridylethylamine (100 μM, 30 s) but not the H2R agonist amthamine (20 μM, 30 s), resulted in a reliable decrease in [Cl^−^]_i_ in a subset of dissociated SCN cells (450 of 1121 cells from 26 mice) (Fig. 1b, left trace); the average peak amplitude of the H1R agonist-induced Cl^−^ response (ΔF) was 0.054 ± 0.003 (*n* = 450). In the remaining cells, H1R agonist produced either an increase (ΔF = -0.069 ± 0.003, *n* = 375) (Fig. 1b, right trace), or no change in [Cl^−^]_i_ (*n* = 296). After bumetanide (10 μM) treatment, the H1R agonist-induced decrease in [Cl^−^]_i_ was converted into an increase [*n* = 81 cells from 4 mice, ΔF = 0.053 ± 0.003 to −0.094 ± 0.003; t(80) = 35.258, *p* < 0.001, paired *t*-test)] while the H1R agonist-induced increase in [Cl^−^]_i_ was enhanced [*n* = 80 cells from 4 mice, ΔF = -0.038 ± 0.002 to −0.094 ± 0.004; t(79) = 15.929, *p* < 0.001, paired *t*-test) (Fig. 1c). Collectively, these results indicate that histamine causes efflux of NKCC1-accumulated Cl^−^ in a subset of SCN neurons.

### H1R-G_βγ_-cAMP/PKA pathway mediates histamine response

**Fig. 2:**
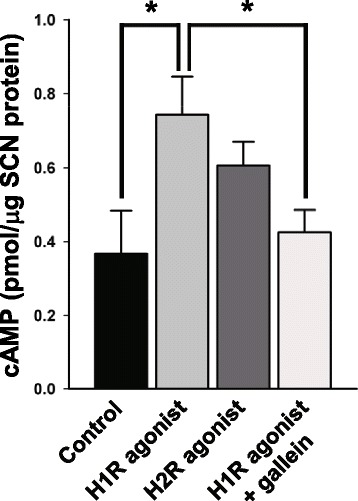
H1R activation increases the cAMP level in the SCN through the G_βγ_ protein. Graph showing the effects on the content of cAMP in the mouse SCN tissue, of H1R (2-pyridylethylamine, 100 μM, *n* = 5 mice) and H2R (amthamine, 20 μM, *n* = 5 mice) agonists alone or in combination with the G_βγ_ inhibitor gallein (100 μM, *n* = 6 mice). Control: *n* = 5 mice. One-way ANOVA followed by Student-Newman-Keuls pairwise comparison test (*: *p* < 0.05)

**Fig. 3:**
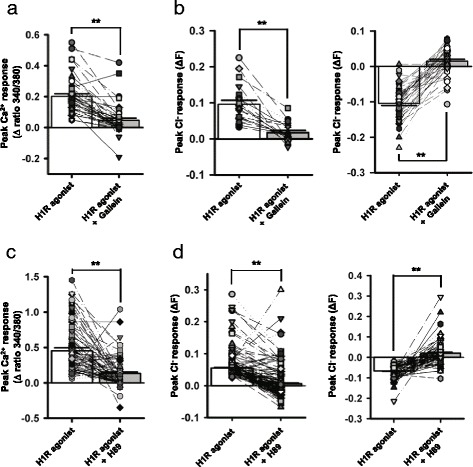
G_βγ_-cAMP/PKA signaling pathway plays a crucial role in H1R agonist-elicited Ca^2+^ rise and Cl^−^ fluxes. **a** Summary graphs showing the effects of the G_βγ_ blocker gallein on H1R agonist-elicited increase in [Ca^**2+**^]_i_. The bar charts indicate the mean (± SEM) peak Ca^**2+**^ responses. **: *p* < 0.001 [*n* = 47 neurons from 3 mice, t(46) = 10.409, paired *t*-test]. **b** Summary graphs showing the effects of gallein on H1R agonist-induced Cl^−^ efflux [*left panel*, *n* = 21 cells from 4 mice, t(20) = 6.783, *p* < 0.001, paired *t*-test] and influx [*right panel*, *n* = 50 cells from 4 mice, t(49) = −14.644, *p* < 0.001, paired *t*-test]. The bar charts indicate the mean (± SEM) peak Cl^−^ responses. **: *p* < 0.001. **c** Summary graphs showing the effects of the PKA inhibitor H89 on H1R agonist-elicited increase in [Ca^2+^]_i_. The bar charts indicate the mean (± SEM) peak Ca^2+^ responses. **: *p* < 0.001 [*n* = 90 neurons from 3 mice, t(89) = 7.272, paired *t*-test]. **d** Summary graphs showing the effects of H89 on H1R agonist-induced Cl^−^ efflux [*left panel*, *n* = 277 cells from 8 mice, t(276) = 19.895, paired *t*-test] and influx [*right panel*, *n* = 90 cells from 8 mice, t(89) = −11.458, paired *t*-test]. The bar charts indicate the mean (± SEM) peak Cl^−^ responses. **: *p* < 0.001. The symbols connected by lines in (**a**-**d**) denote data from the same cells

Previous work suggests that H1R is positively linked to cAMP signaling pathway through the G_βγ_ protein [18, 19], and it is well known that cAMP is a strong PKA activator [57, 58]. Thus, we next explored the possibility that the G_βγ_ protein arising from the activation of H1R promotes the cAMP production to activate PKA and ultimately to bring about histamine-induced responses in SCN neurons. To this end, we examined whether H1R activation leads to the increase in cAMP level in the SCN and, if so, the treatment of gallein (G_βγ_ protein inhibitor) blocks this increase, and whether gallein and the PKA blocker H89 suppress the H1R agonist-elicited Ca^2+^ and Cl^−^ responses in dissociated SCN cells. We found that application of the H1R agonist 2-pyridylethylamine (100 μM, for 30 min starting at ZT 14:00 h), but not the H2R agonist amthamine (20 μM, for 30 min starting at ZT 14:00 h), resulted in a significant increase in cAMP content in the SCN and this effect was precluded by co-applied gallein (100 μM) [one-way ANOVA: F(3,17) = 3.828, *p* = 0.029, Student-Newman-Keuls pairwise comparison tests: Control *vs.* H1R agonist: *p* = 0.037, Control *vs*. H2R agonist: *p* = 0.170; Control *vs*. H1R agonist + gallein: *p* = 0.635; H1R agonist *vs*. H2R agonist: *p* = 0.289; H1R agonist *vs*. H1R agonist + gallein: *p* = 0.043; H2R agonist *vs*. H1R agonist + gallein: *p* = 0.153] (Fig. 2). Moreover, we discovered that both gallein (100 μM) and H89 (10 μM) generally suppressed the H1R agonist (100 μM, 30 s)-elicited Ca^2+^ and Cl^−^ responses (Fig. 3). Thus, taken together, these results suggested that the H1R-G_βγ_-cAMP/PKA pathway mediates histamine-elicited responses in SCN neurons.

### CFTR mediates histamine-induced Cl^−^ efflux

**Fig. 4:**
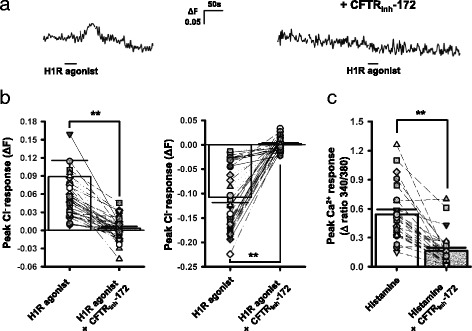
The CFTR inhibitor CFTR_inh_-172 suppresses histamine-elicited Cl^−^ efflux and Ca^2+^ rise in SCN cells. **a** Traces from Cl^−^-imaging experiments that show the inhibitory effects of CFTR_inh_-172 on 2-pyridylethylamine (H1R agonist)-induced Cl^−^ efflux in an SCN cell. **b** Summary graph showing the effect of CFTR_inh_-172 on H1R agonist-induced Cl^−^ efflux (*n* = 41 cells from 4 mice; *left panel*) and influx (*n* = 38 cells from 4 mice; *right panel*) in SCN cells. **c** Summary graph showing the effect of CFTR_inh_-172 on histamine-elicited increase in [Ca^2+^]_i_ in SCN neurons (*n* = 31 neurons from 3 mice). The bar charts in (**b**) and (**c**), respectively, indicate the mean (± SEM) Cl^−^ and Ca^2+^ responses elicited by H1R agonist or histamine in the absence or presence of CFTR_inh_-172. The symbols connected by lines in (**b**) and (**c**) denote data from the same cells. **: *p* < 0.001

In order to better understand the histaminergic regulation of Cl^−^ in SCN neurons, we then explored the possible role of CFTR, the PKA-activated Cl^−^ channel. Specifically, we examined the effects of the CFTR inhibitor CFTR_inh_-172 on histamine (or H1R agonist)-induced responses in SCN cells. We found that the application of CFTR_inh_-172 (50 μM) blocked both Cl^−^ efflux [ΔF: 0.111 ± 0.035 to 0.003 ± 0.003, *n* = 41 cells from 4 mice; W = -861, *p* < 0.001, Wilcoxon Signed Rank test; Fig. 4a and b, left panel] and influx [ΔF: −0.108 ± 0.011 to 0.002 ± 0.002, *n* = 38 cells from 4 mice; W = 741, *p* < 0.001, Wilcoxon Signed Rank test; Fig. 4b, right panel] induced by 2-pyridylethylamine (H1R agonist; 100 μM, 30 s) in SCN cells. Furthermore, we discovered that CFTR_inh_-172 attenuated significantly the histamine (100 μM, 30 s)-elicited Ca^2+^ rise in SCN neurons [0.53 ± 0.05 to 0.15 ± 0.03, *n* = 31 cells from 3 mice; t(30) = 6.849, *p* < 0.001, paired *t*-test] (Fig. 4c). Collectively, these results indicated that CFTR mediates histamine-induced Cl^−^ fluxes in SCN neurons.

### Histamine resets the circadian clock through the G_βγ_-cAMP/PKA-CFTR pathway

**Fig. 5:**
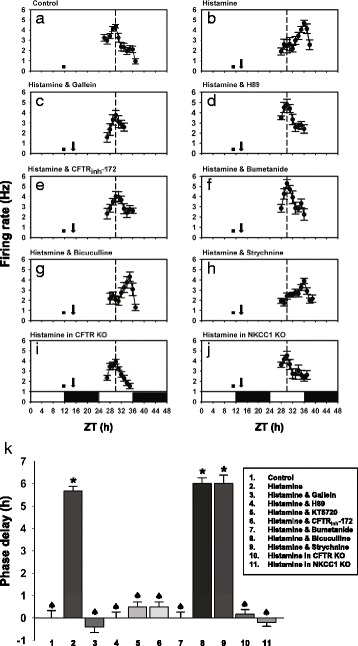
Effect of blockade/KO of G_βγ_, PKA, CFTR or NKCC1 on histamine-induced resetting of circadian clock. **a**-**j** Plots against ZT of the firing rate of SCN neurons recorded in different experimental conditions. Each plot shows the representative result of 6 repeated experiments. The projected light and dark phases of the animal room are indicated with open and filled horizontal bars, respectively. The dashed vertical line in each plot indicates the average time of peak neural activity for control slices. The filled square denotes the time of slice preparation, while the arrow indicates the time of drug application. **k** Graph summarizing the effects of various experimental treatments on the time of peak of circadian firing activity rhythm. Student-Newman-Keuls comparison tests were performed after Kruskal-Wallis one-way ANOVA on Ranks (*p* < 0.001). The results of pair-wise comparisons of the value of each experimental group with those of control and histamine groups are denoted with asterisk and spade, respectively. *, ♠: *p* < 0.05

**Fig. 6:**
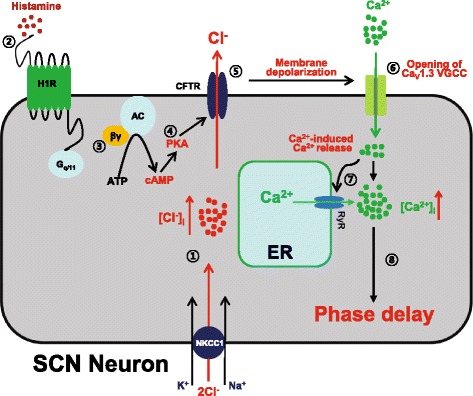
Proposed signaling pathway for histamine-induced resetting of the circadian pacemaker in the SCN. ① A subset of SCN neurons are loaded with Cl^−^ by the action of the Cl^−^ importer NKCC1. ② H1R stimulation by histamine at early night results in the production of activated G_βγ_ protein in these cells. ③ The activated G_βγ_ protein stimulates adenylate cyclase (AC) to produce cAMP from ATP. ④ PKA activated by cAMP opens the Cl^−^ channel CFTR. ⑤ The efflux of Cl^−^ through CFTR down the electrochemical gradient results in membrane depolarization. ⑥ In response to this membrane depolarization, the Ca_V_1.3 L-type voltage-gated Ca^2+^ channel (VGCC) is activated. ⑦ The resulting Ca^2+^ influx through Ca_V_1.3 VGCC induces Ca^2+^ release from the endoplasmic reticulum (ER) through RyR. ⑧ The consequent increase in [Ca^2+^]_i_ leads to phase delay of the circadian clock

Our previous work has indicated that, in order to reset the circadian clock, histamine raises [Ca^2+^]_i_ in SCN neurons by activating the Ca_V_1.3 L-type Ca^2+^ channels through H1R, and secondarily by causing Ca^2+^-induced Ca^2+^ release from RyR-mediated internal stores [12]. Meanwhile, the results described above suggest that the G_βγ_-cAMP/PKA-CFTR pathway is responsible for linking H1R to L-type Ca^2+^ channel. To further test this hypothesis, we investigated how the pharmacological blockade or genetic removal of G_βγ_, PKA, CFTR or NKCC1 altered the histamine-elicited phase delay of circadian neural activity rhythm in the mouse SCN slice. In the control slices, the peak of the circadian neural activity rhythm occurred at ZT 6.00 ± 0.33 h (*n* = 6; Fig. 5a and k). In the histamine (100 μM)-treated slices, the peak was phase-delayed about 6 h to ZT 11.67 ± 0.21 h (*n* = 6; Fig. 5b and k) by the next cycle [6, 12]. This histamine effect on the circadian phase was almost completely blocked by the co-applied inhibitors of G_βγ_ (gallein, 100 μM), PKA (H89, 10 μM; KT5720, 1 μM), CFTR (CFTR_inh_-172, 50 μM) and NKCC (bumetanide, 10 μM) (Fig. 5c-f and k), but not by the GABA_A_ receptor antagonist bicuculline (30 μM) or the glycine receptor antagonist strychnine (1 μM) (Fig. 5g, h and k). In addition, the histamine-elicited phase delays were absent in KO mice lacking CFTR and NKCC1 (Fig. 5i-k). In control experiments, we found that application of the blockers alone or the genetic loss of CFTR and NKCC1 did not alter the phase of the circadian rhythm. The time of peak electrical activity of SCN neurons recorded in the slices treated with either gallein [ZT 6.5 ± 0.3 h, *n* = 4; *p* = 0.242, t(8) = −1.265, *t*-test], H89 [ZT 5.8 ± 0.3 h, *n* = 4; *p* = 0.610, U = 14.5, Mann–Whitney Rank Sum Test], CFTR_inh_-172 [ZT 5.5 ± 0.3 h, *n* = 4; *p* = 0.242, t(8) = 1.265, *t*-test] or bumetanide alone [ZT 6.5 ± 0.3 h, *n* = 4; *p* = 0.242, t(8) = −1.265, *t*-test] was not significantly different from that of neurons recorded in drug-untreated, control slices (ZT 6.00 ± 0.33 h, *n* = 6). Likewise, the time of peak electrical activity of SCN neurons recorded in the slices from CFTR KO [ZT 6.3 ± 0.5 h, *n* = 4; *p >* 0.999, U = 12, Mann–Whitney Rank Sum test] or NKCC1 KO mice [ZT 6.0 ± 0.4 h, *n* = 4; *p* > 0.999, *U* = 12, Mann–Whitney Rank Sum Test] was not significantly different from that of neurons recorded in the slices from wild-type animals (ZT 6.00 ± 0.33 h, *n* = 6). Thus, taken together, the results from the functional assay further supported our hypothesis that the G_βγ_-cAMP/PKA-CFTR pathway links H1R to Ca_V_1.3 L-type Ca^2+^ channels in SCN neurons, leading to the resetting of the circadian clock (Fig. 6).

## Discussion

In a recent communication [12] we reported that, in order to reset the circadian clock in the mouse SCN, histamine increases [Ca^2+^]_i_ in SCN neurons by activating the Ca_V_1.3 L-type Ca^2+^ channels through H1R, and secondarily by causing Ca^2+^-induced Ca^2+^ release from RyR-mediated internal stores. Moreover, we provided evidence that IP_3_ and DAG, the downstream of PLC, are not involved in the H1R-mediated rise in [Ca^2+^]_i_. In the current study, we explored the signaling mechanism that links the H1R to the Ca_V_1.3 channels and obtained the results indicating that, in a subset of SCN neurons loaded with Cl^−^ by NKCC1, histamine causes Cl^−^ efflux through H1R to produce membrane depolarization needed for the activation of Ca_V_1.3 channels. CFTR is well known as anion channel whose malfunction leads to dysregulation of epithelial fluid transport in the lung, pancreas and other organs, resulting in cystic fibrosis [22, 24, 59]. In this study, we present evidence that CFTR mediates the histamine-elicited Cl^−^ efflux in SCN cells while the G_βγ_-cAMP/PKA pathway downstream of H1R activates the CFTR. Studies have provided evidence that histamine-activated Cl^−^ channels are expressed in mammalian brain [13, 14] and that CFTR mRNA exists in the mouse SCN ([17], http://www.brain-map.org). Also, it has been reported that H1R is positively coupled through the G_βγ_ protein to the cAMP signaling pathway [18, 19], which can activate CFTR by up-regulating PKA [20–24]. Nevertheless, the H1R-mediated signaling pathway that involves CFTR has never been reported. To the best of our knowledge, the current study is the first to provide the evidence for the existence of such a signaling pathway. Also, this study is the first to present evidence indicating that CFTR has a functional role in the circadian time-keeping system.

In the current study we noticed that, even after the blockade of CFTR, a significant proportion of SCN neurons still responded to histamine with an increase in [Ca^2+^]_i_ although the magnitudes of the responses were significantly smaller. This finding suggests that mechanism(s) other than the membrane depolarization arising from CFTR-mediated Cl^−^ efflux also contributes to the L-type Ca^2+^ channel activation by histamine. The possible mechanisms include the direct activation of the Ca^2+^ channel by PKA [60–63] and the blockade of leak K^+^ channels through the H1R-coupled G_q/11_ protein [2], which can produce the membrane depolarization needed for the activation of the Ca^2+^ channels. Whether these mechanisms operate in SCN neurons for the histaminergic activation of L-type Ca^2+^ channels and, if so, whether they work along with the “CFTR” mechanism in the same cells await further investigation.

The results of the current study indicate that NKCC1 is crucial for the histamine-elicited increase in [Ca^2+^]_i_ in SCN neurons and the subsequent resetting of the circadian clock in the SCN. By importing Cl^−^, NKCC1 provides the driving force for the histamine-elicited Cl^−^ efflux through CFTR which produces membrane depolarization needed for the activation of L-type Ca^2+^ channels. Our data do not suggest that histamine effects depend on GABAergic and/or glycinergic transmission, the polarity and strength of the postsynaptic responses of which can be regulated by NKCC1. This view is consistent with the finding that histamine caused [Ca^2+^]_i_ to rise in dissociated SCN neurons which were no longer under synaptic control. Moreover, it is consistent with the observation that both bicuculline and strychnine had no significant effects on the histamine-elicited phase delay of the circadian neural activity rhythm in the SCN slice.

The present study demonstrates that, in certain SCN cells (~33 % cells examined), H1R activation led to Cl^−^ influx, instead of efflux. This is presumably because of that, in these cells, the NKCC1 activity was low or absent such that the electrochemical gradient for Cl^−^ was set toward the intracellular side. At present, we do not know the functional significance of the histamine-induced Cl^−^ influx. However, it is unlikely that this Cl^−^ flux promoted histamine to phase-delay the circadian pacemaker in the SCN, since the blockade of NKCC1 by bumetanide, which converted the H1R agonist-induced Cl^−^ efflux into influx in some cells and enhanced the H1R agonist-induced Cl^−^ influx in other cells, resulted in a complete blockade of the histamine-elicited phase shift.

### Significance

A variety of evidence indicates that histamine is a potent regulator of the phase of the circadian system. Prior work has shown that histamine application onto SCN neurons in the rat and hamster hypothalamic slices increases or decreases the electrical activities of these cells [64–66] while bath-applied histamine phase-shifts the circadian neural activity rhythms recorded in hamster and mouse SCN slices [6, 7, 27]. The inhibition of histamine synthesis by α-fluoromethylhistidine decreases the phase shifts of circadian activity rhythms induced by light in the hamster [9]. Injection of histamine into the cerebral ventricle in the rat leads to the phase shifts of circadian activity rhythms [8] and decreases the time needed to entrain activity rhythms to an abruptly advanced light–dark cycle [67]. In our own work, we demonstrated that histamine acts on H1R to alter the phase of the circadian clock in the mouse SCN and this effect depends on the increase of [Ca^2+^]_i_ which results from the activation of Ca_V_1.3 L-type Ca^2+^ channels and Ca^2+^-induced Ca^2+^ release from RyR-mediated internal stores [12]. In the present study, we further explored the signal transduction mechanisms and present evidence that the G_βγ_-cAMP/PKA-CFTR pathway is intercalated between H1R and the L-type Ca^2+^ channels. These results clearly delineate a novel signaling mechanism that underlies the histamine-induced resetting of the circadian clock.

More broadly, there are many remaining questions about the role of histamine in the generation of circadian rhythms *in vivo* and the functional significance of the phase control remains unclear. Among the complications is evidence for species-specific differences as Scott et al. [66] showed that the hamster SCN is almost devoid of histaminergic fibers and that histamine has only a weak phase-shifting effect on the free-running locomotor activity rhythm. In addition, the inhibition of histamine synthesis by α-fluoromethylhistidine or the KO of histidine decarboxylase gene disrupted the circadian activity rhythm in the rat [8] and lowered the levels of wheel-running and spontaneous locomotor activities in mice in both light–dark and dark-dark conditions [68]. These findings raise the possibility that histamine may be involved in the generation of the rhythms as well as phase control. The receptor mediating these effects is less clear. Studies on H1R KO mice found disrupted diurnal feeding rhythm [69] but essentially normal locomotor activity rhythms [70]. In contrast, the H3R KO mice exhibited a greatly reduced locomotor activity rhythm but normal rhythms in clock gene expression [70]. Unfortunately, the studies with the KO mice did not examine whether these genetic manipulations altered the photic regulation of the circadian cycle. Taken together, these data indicate that histamine is a potent regulator of the circadian time-keeping system in some mammals and suggest that the H1R signaling is important for phase control while the H3R signaling may be more important for regulation of the amplitude of circadian rhythms in activity.

## Conclusions

In summary, we present experimental results indicating that, in order to activate Ca 1.3 L-type Ca channels in SCN neurons and ultimately to phasedelay the circadian clock in the SCN, histamine recruits the H1R-G -cAMP/PKACFTR pathway in the SCN neurons loaded with Cl by NKCC1. This pathway and NKCC1 may well be potential targets for drugs designed to treat clinical problems resulting from the disturbance of the circadian system.

### Ethics approval

The experimental procedures employed in the present study were approved by the Animal Research Policies Committees of Korea University College of Medicine and Korea Institute of Science and Technology.

### Consent for publication

Not applicable.

### Availability of data and materials

We do not have any special data or materials to share with readers.

## Abbreviations

[Ca^2+^]_i_: intracellular calcium concentration
[Cl^−^]_i_: intracellular chloride concentration
ACSF: artificial cerebrospinal fluid
ANOVA: one-way analysis of variance
CFTR: cystic fibrosis transmembrane conductance regulator
DAG: diacyl-glycerol
H1R: histamine 1 receptor
IP_3_: Inositol 1,4,5-trisphosphate
KO: knock-out
MQAE: N-(ethoxy carbonyl methyl)-6-methoxyquinolinium bromide
NKCC1: Na^+^-K^+^-2Cl^−^ cotransporter isotype 1
PKA: protein kinase A
PLC: phospholapase C
R/T: room temperature
SCN: suprachiasmatic nucleus
RyR: ryanodine receptor
TMN: tuberomammillary nucleus
TRPC: transient receptor potential cation channel
VGCC: voltage-gated calcium channel
ZT: zeitgeber time
